# Egr2 targets identify a population of dysfunctional T cells in the tumor microenvironment with immune modulatory properties

**DOI:** 10.1186/2051-1426-2-S3-P238

**Published:** 2014-11-06

**Authors:** Jason Williams, Yan Zheng, Thomas Gajewski

**Affiliations:** 1 University of Chicago, Chicago, IL, USA

## 

Although the presence of tumor-infiltrating lymphocytes (TILs) indicates an endogenous anti-tumor response, immune regulatory pathways can subvert the effector phase and enable tumor escape. Negative regulatory pathways include expression of inhibitory receptors and corresponding ligands, metabolic dysregulation, recruitment of suppressive cell populations, and T cell-intrinsic anergy. Recently, we have shown that the transcription factor Egr2 is critical in controlling the anergic state using an *in vitro *model system. Gene expression profiling and Egr2 ChIP-Seq analysis revealed multiple Egr2-driven cell surface proteins in T cell anergy, including the inhibitory receptor Lag3, but also the costimulatory receptor 4-1BB. We examined whether these surface proteins might be useful for identifying the dysfunctional tumor-reactive CD8^+ ^T cells within the tumor microenvironment. Flow cytometric analysis of TIL in the B16, C1498, and MC57 tumor models revealed a major population of CD8^+ ^T cells expressing Lag3 along with the defined inhibitory receptor PD-1. Approximately 43% of these cells expressed 4-1BB, and kinetic analysis showed appearance of this population in the tumor over time. Cell sorting revealed that the Lag3/PD-1/4-1BB-triple positive cells showed the most severe dysfunction as reflected by defective IL-2 production. qRT-PCR confirmed expression of multiple anergy-associated genes, including Egr2. Using an Egr2/IRES-GFP knock-in mouse, Egr2GFP^high ^TILS were confirmed to show defective IL-2 production ex vivo. To examine whether this CD8^+ ^T cell population was likely to represent those recognizing specific antigens, TCR repertoire analysis was performed and indicated TCRβ skewing in the marker positive population, indicating oligoclonality. Analysis of CD8^+ ^TIL specific for the model antigen SIY confirmed that the vast majority of these cells expressed Lag3, PD-1, and 4-1BB. Despite the inability to make IL-2, the Lag3/PD-1/4-1BB-triple positive cells produced high levels of IL-10, IFN-g, CCL1, and CCL22, arguing that they might exert immunomodulatory functions and not be completely inert. Indeed, an *in vitro *suppression assay revealed that these cells are capable of inhibiting proliferation of conventional T cells with a potency similar to that seen with FoxP3^+ ^regulatory T cells. Our results suggest that the co-expression of Lag3, PD-1, 4-1BB, and perhaps Egr2 itself may identify a critical subpopulation of dysfunctional TILs that are specific for tumor antigens and contribute to an immune suppressive tumor microenvironment. Ultimately, inhibiting or agonizing receptors on this subpopulation could have therapeutic relevance.

